# Confirmatory Factor Analysis of the Geriatric Depression Scale to Measure Apathy in Older Adults

**DOI:** 10.1093/geroni/igaa057.1187

**Published:** 2020-12-16

**Authors:** Mirnova Ceide, Aviva Cantor, Joe Verghese, David Lounsbury

**Affiliations:** Albert Einstein College of Medicine, Bronx, New York, United States

## Abstract

Apathy is a psychological syndrome characterized by lack of motivation, interest, flattening of affect and social withdrawal; which has been associated with cognitive and functional decline in community dwelling older adults. Many of these associations have been investigated using subscales of depression tools, with varying sensitivity and specificity. The objective of this study was to develop an apathy measurement model, which distinguishes depression and apathy, using the Geriatric Depression Scale Long Form (GDS). We conducted an exploratory factor analysis (EFA) in a memory clinic cohort (N=81) who completed the GDS. Based on the EFA results, we ran a confirmatory factor analysis (CFA) in a community dwelling cohort of older adults (≥65 years old) enrolled in the Central Control of Mobility in Aging study (CCMA) and computed factor scores in both cohorts. CFA of the GDS in the CCMA cohort (N=538) yielded 3 factors: Depression (14 items; Cronbach’s α=0.77), Apathy (10 items; Cronbach’s α=0.63), and Cognitive concern (4 items; Cronbach’s α=0.546). In linear regression models, adjusted for age and gender, the memory clinic cohort had significantly higher Apathy factor scores (β = .18, t(618) = 4.49, p < .001) factor scores compared to the CCMA cohort.Our findings suggest that the CFA derived apathy score is a reliable and valid tool to distinguish apathy from depression in older adults. This novel measurement model can be employed in both clinic and research populations to investigate the role of apathy in cognitive decline.

